# Fabrication of a Miniaturized ZnO Nanowire Accelerometer and Its Performance Tests

**DOI:** 10.3390/s16091499

**Published:** 2016-09-14

**Authors:** Hyun Chan Kim, Sangho Song, Jaehwan Kim

**Affiliations:** Creative Research Center for Nanocellulose Future Composites, Department of Mechanical Engineering, Inha University, 100 Inha-Ro, Nam-Ku, Incheon 22212, Korea; kim_hyunchan@naver.com (H.C.K.); whysosary@naver.com (S.S.)

**Keywords:** accelerometer, ZnO nanowire, miniaturization, piezoelectric, micro fabrication

## Abstract

This paper reports a miniaturized piezoelectric accelerometer suitable for a small haptic actuator array. The accelerometer is made with zinc oxide (ZnO) nanowire (NW) grown on a copper wafer by a hydrothermal process. The size of the accelerometer is 1.5 × 1.5 mm^2^, thus fitting the 1.8 × 1.8 mm^2^ haptic actuator array cell. The detailed fabrication process of the miniaturized accelerometer is illustrated. Performance evaluation of the fabricated accelerometer is conducted by comparing it with a commercial piezoelectric accelerometer. The output current of the fabricated accelerometer increases linearly with the acceleration. The miniaturized ZnO NW accelerometer is feasible for acceleration measurement of small and lightweight devices.

## 1. Introduction

**Figure 1:**
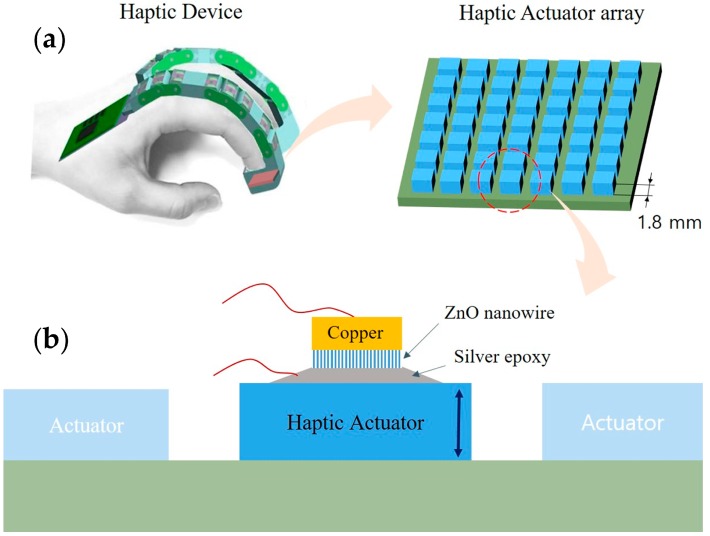
Concept of a miniaturized ZnO NW accelerometer: (**a**) Haptic device for a simulator and the haptic actuator array; (**b**) Miniaturized ZnO NW accelerometer attached on the cell of the haptic actuator array.

As simulator technologies have evolved, haptic actuators have become required to display realistic tactility for simulators. Simulators require transferring visual information in conjunction with haptic information to users [1,2]. Haptic devices that carry haptic information are essential for simulators. One of the important kinds of haptic information for effective perception is the tactile sensation that obtains sensory data from skin receptors. In order to create a variety of tactile sensations, it is necessary to use vibrotactile actuators that can vibrate in a wide frequency range [3]. Figure 1a shows the concept of a haptic device for a simulator on which haptic actuator array is installed. The cells in the haptic actuator array generate individual vibrations so as to mimic the tactile sensation. The size of haptic actuator array is 7 × 7 array with 15 mm × 15 mm overall size. The cell size of the array is 1.8 mm × 1.8 mm.

To demonstrate the haptic actuator array, its acceleration at each cell of the array should be evaluated. There are non-contact and contact type approaches to measure acceleration. Among the non-contact type, laser Doppler velocimeters (LDVs) are popular since they can precisely measure velocity and give an acceleration value, but LDVs are so bulky and expensive that they are not suitable for on-site acceleration measurement in a haptic actuator array. Among the contact type, piezoelectric accelerometers can be attached to any kind of structure and actuator to detect acceleration [4], but conventional piezoelectric accelerometers are too heavy to be attached to the haptic actuator array. Thus, a targeted piezoelectric accelerometer should be miniaturized to match the size of the small haptic actuator array.

The piezoelectric accelerometer uses the direct piezoelectric effect which converts mechanical vibration energy into electrical energy. There are three types of piezoelectric accelerometers: compression, shear, and bending types [5]. Among them, the compression type offers a simple structure, high rigidity, high resonance frequency, and broad response range [6]. Compression types consist of a piezoelectric material sandwiched between a proof mass and the mounting base. When the compression type piezoelectric accelerometer vibrates vertically, the inertia of the proof mass exerts a force on the piezoelectric material. The seismic mass and the stiffness of the piezoelectric material is important, since the material should have enough sensitivity along with good mechanical stiffness and miniaturization.

For the piezoelectric material, ZnO NW was chosen. ZnO NW is a well-known nanomaterial used for nanocomposites and nanogenerators [7,8]. Furthermore, recently the ZnO NW is drawing attention for energy harvesting and sensor applications [8,9,10,11,12]. The wurtzite structure of ZnO NW exhibits piezoelectricity like a non-ferroelectric piezoelectric material. It has high aspect ratio, which leads to large deformation and high piezoelectric response output. Using ZnO NW as active material has advantages of applicability thanks to its simple structure, light weight, low unit cost and environmentally friendliness, unlike lead-based piezoelectric ceramics. ZnO NW can be fabricated using various methods—metalorganic chemical vapor deposition, pulsed laser deposition, physical vapor deposition, electro deposition and hydrothermal synthesis [13,14,15,16,17]. Since the hydrothermal synthesis process does not require the use of high vacuum and temperature conditions, it is inexpensive and has a possibility of mass production. It also allows one to control the ZnO NW growth size under diverse conditions of the process on different kinds of substrates [18,19].

The objective of this paper is miniaturization of a ZnO NW-based accelerometer and its performance test in a haptic actuator array. The size of the accelerometer is 1.5 × 1.5 mm^2^ that fits onto the 1.8 × 1.8 mm^2^ cell of the haptic actuator array. Figure 1b illustrates the accelerometer attached on the cell. The haptic actuator array generates vibrations in the frequency range of 50~500 Hz with 1 g maximum acceleration (g = 9.81 m/s^2^). Thus, the accelerometer should be able to measure accelerations between 50 and 500 Hz up to 1 g acceleration level. This paper illustrates how to design the configuration of the accelerometer with that specification in conjunction with the fabrication and performance evaluation.

## 2. Design

The accelerometer consists of a proof mass and the stiffness of the piezoelectric layer, so in the design of the accelerometer, determination of the mass and stiffness is important. The first criterion is the mass design. The mass of the accelerometer should be less than 1/10 the mass of the vibration actuator because the acceleration of the vibration actuator is reduced after installation of accelerometer by an amount given by m_1_/(m_1_ + m_2_), where m_1_ is mass of the vibration actuator and m_2_ is the mass of the accelerometer [20]. In the fabricated accelerometer, the proof mass mainly governs the accelerometer mass. The mass of a single actuator cell is approximately 0.41 gram. Thus, the proof mass of the accelerometer is set to 0.04 gram. Copper was chosen for the proof mass because of its heavy density. For a 0.04 gram proof mass, the dimensions of Cu should be 1.5 × 1.5 × 2 mm^3^. The second criterion is the stiffness design. The stiffness (k) of the ZnO NW array is equal to *AE/l*, where *A* is cross sectional area , *E* is Young’s modulus of the ZnO NW and *l* is thickness of the ZnO NW. When the thickness of ZnO NW is 8 µm and *E* = 21 GPa [21], the stiffness of the ZnO NW is approximately 6 × 10^8^ N/m. Furthermore, with 0.04 g of the proof mass (m), the fundamental resonance of the fabricated accelerometer is $\omega =\sqrt{k/m}=616\;\mathrm{rad}/s$. As a result, it can have 205 kHz of cut-off frequency by f=(1/2π)ω. Thus, it can measure accelerations between 50 and 500 Hz.

## 3. Experiments

### 3.1. Fabrication

**Figure 2:**
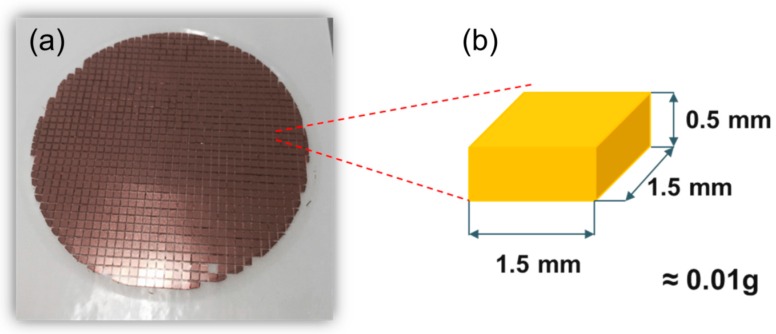
Cu wafer: (**a**) polished image and (**b**) size of diced one.

Figure 2 shows a polished Cu wafer (with thickness of 500 µm) used for the proof mass in this work. The polished Cu wafer was diced into 1.5 mm × 1.5 mm by a diamond blade dicing machine. The mass of one cell is approximately 0.01 g. The dicing area determines the size of the miniaturized accelerometer.

**Figure 3:**
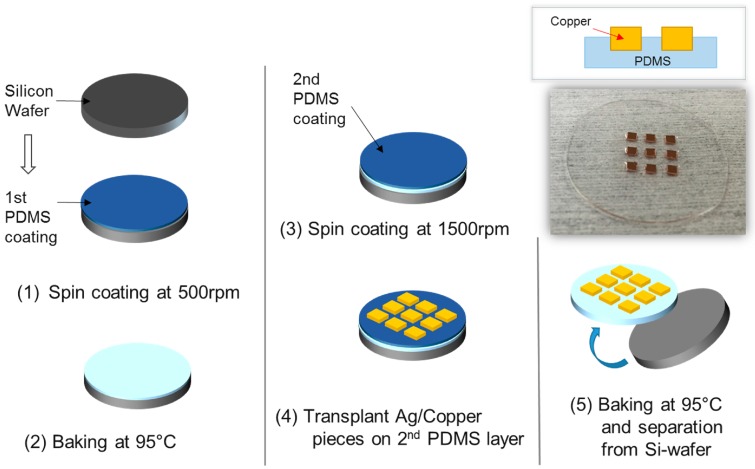
Fabrication process of the PDMS boat.

On the surface of the diced Cu substrate, ZnO NW should be grown by a hydrothermal process. A polydimethylsiloxane (PDMS, Sylgard^®^ 184) boat is necessary to hold the Cu substrates face-down, floating on top of the solution, so as to protect the precipitation of ZnO on the surface during the hydrothermal reaction. Figure 3 shows the overall fabrication process of the PDMS boat and attachment of the Cu substrate on it. The PDMS boat was produced by a spin coating and baking process. Firstly, the PDMS solution, the mixture of the elastomer base, was mixed with a hardening agent at the ratio of 10:1, and then coated on a silicon dummy wafer at 500 rpm, followed by baking at 95 °C for 10 min. After the first PDMS coating, additional PDMS spincoating was performed on it at 1500 rpm. Then the Cu substrate was transplanted onto the PDMS layer, maintaining the polished surface of the Cu substrates shown above the PDMS layer. The polished surface should be accessible in order for the ZnO NW growth reaction to occur. Further baking at 95 °C for 10 min was carried out for curing, and fixing the PDMS layer. By separating the PDMS layer from the silicon wafer, the fabrication of the boat was completed.

**Figure 4:**
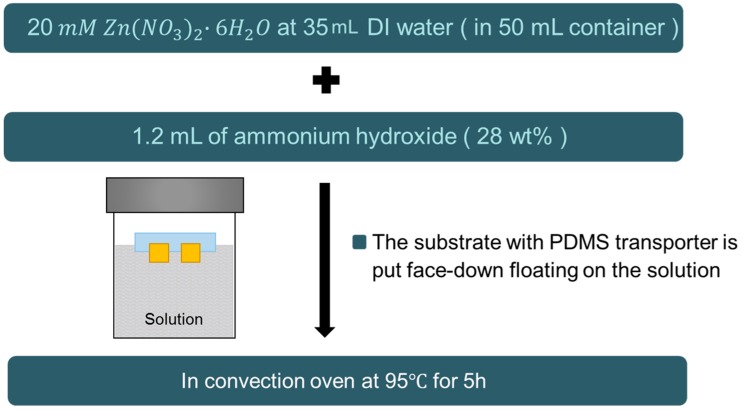
Reaction process of the hydrothermal synthesis of ZnO NW.

The hydrothermal synthesis process of the ZnO NW is shown in Figure 4. ZnO NWs were deposited on the Cu substrate as the active piezoelectric layer of the accelerometer by using the hydrothermal synthesis method [17]. For the hydrothermal synthesis process, a nutrient solution was used by mixing two precursors—zinc nitrate hexahydrate and ammonium hydroxide. In this work, 1.2 mL ammonium hydroxide was added in the 35 mL of 20 mM zinc nitrate hexahydrate. Then a 50 mL autoclave was filled with the nutrient solution and the PDMS boat of which Cu substrate transplanted, was placed face-down on top of the solution. A strong sealing was used to withstand the internal pressure, thereby getting a high quality ZnO NW. The reaction process was conducted in a convection oven at 95 °C for 5 h.

The Cu substrates on which ZnO NWs were grown were pulled out from the boat and rinsed with deionized water. After drying at room temperature, an extra proof mass was attached on top of the Cu substrate using a silver paste. The extra proof mass was a piece of diced Cu wafer with the same size, 1.5 mm × 1.5 mm, to reach the designed mass. Note that the proof mass functions as a top electrode of the accelerometer at the same time. To mount the fabricated ZnO NW accelerometer on a vibrating surface, silver epoxy was used. A thin silver epoxy layer was deposited on the vibrating surface and the fabricated accelerometer was attached on it. The silver epoxy layer functions as the bottom electrode of the accelerometer.

### 3.2. Performance Test

**Figure 5:**
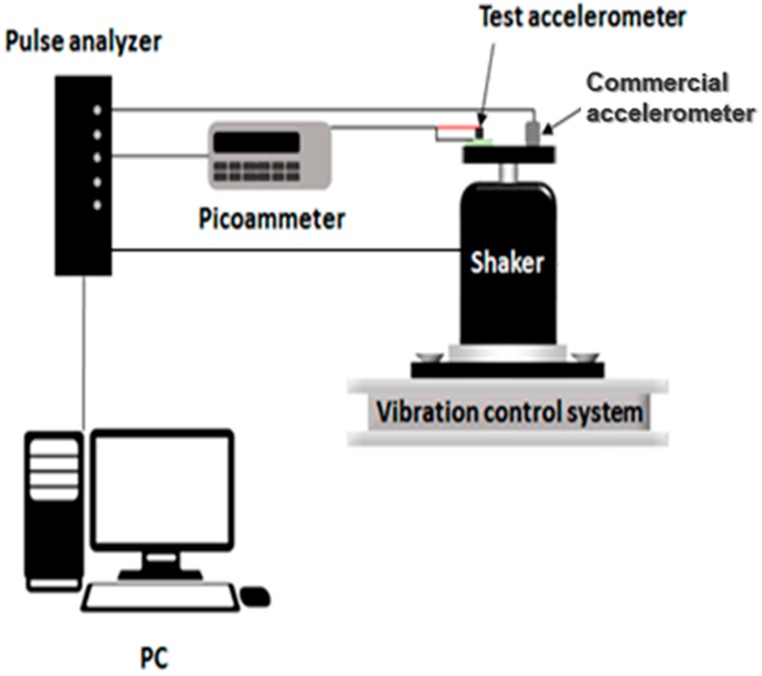
Schematic diagram of the experimental setup for the performance tests.

Performance evaluation of the fabricated ZnO NW accelerometer was conducted by comparing it with a commercial piezoelectric accelerometer (352C33, PCB, Depew, NY, USA). Figure 5 shows the performance test setup. The ZnO NW accelerometer was mounted on the top surface of a shaker, on which the commercial accelerometer attached beside of the fabricated accelerometer. The shaker generates vibrations like the haptic actuator array as an acceleration source. A picoammeter (6485, KEITHLEY, Beaverton, OR, USA) was used to receive current signal from the tested accelerometer. The picoammeter has 10 fA resolution and 20 fA of typical RMS noise. Then a pulse analyzer (35360B-030, Bruel & Kjaer, Naerum, Denmark ) was used to analyze the signals from the fabricated sensor and the commercial accelerometer through BNC cables. Moreover, the pulse analyzer includes a function generator for control of the shaker acceleration by changing the input voltage and frequency.

## 4. Results and Discussion

**Figure 6:**
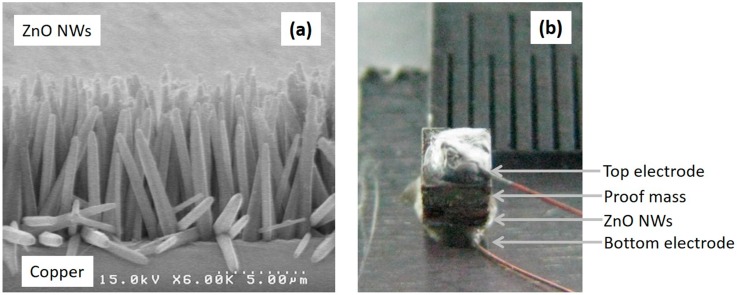
SEM images: (**a**) the grown ZnO NWs; (**b**) the fabricated ZnO NW accelerometer.

In this work, the ZnO NWs were analyzed using a scanning electron microscope (SEM). Figure 6 shows the morphology of the ZnO NWs grown on the Cu substrate. 

The length and diameter of ZnO NWs are approximately 8 µm and 500 nm, respectively. Its high aspect ratio results in improved piezoelectricity. Figure 6b shows a photograph of the fabricated accelerometer. The grown ZnO NWs were bonded on the structure by using a silver epoxy and formed the bottom electrode. The top electrode was made by using the silver epoxy on the copper proof mass.

**Figure 7:**
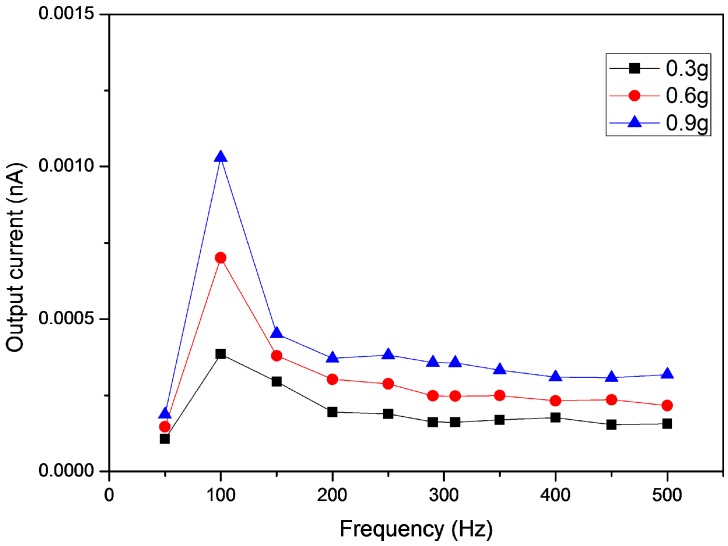
Output current of the fabricated accelerometer with acceleration change.

The performance test of the fabricated accelerometer was performed in the frequency range of 50~500 Hz with 50 Hz step. Figure 7 shows the output current of the fabricated accelerometer with different acceleration levels. Every output current values were collected at three acceleration levels: −0.3, 0.6 and 0.9 g. The form of the measured output signal is a sine wave-like input signal. The reported values for the current are root mean square (RMS) values. The output current increased in proportion to the acceleration change in the operating frequency range. High current output was observed at 100 Hz for the fabricated accelerometer. Note that 100 Hz may be associated with the resonance frequency of the excitation system.

**Figure 8:**
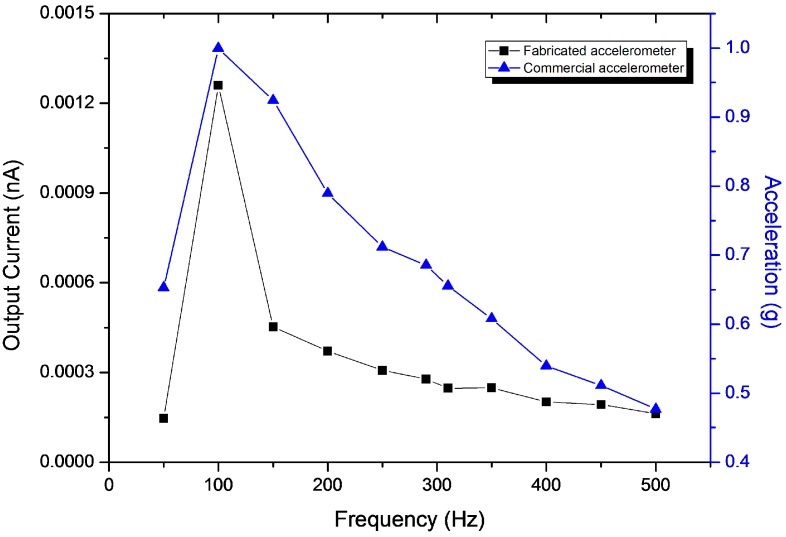
Performance comparison of the fabricated and commercial accelerometers.

Figure 8 shows a comparison of the current output from the fabricated accelerometer and the acceleration value measured from the commercial accelerometer with the frequency sweep. A steady voltage was applied to the shaker and the operating frequency was changed from 50 to 500 Hz. The peak at 100 Hz is a resonance frequency of the excitation system.

**Figure 9:**
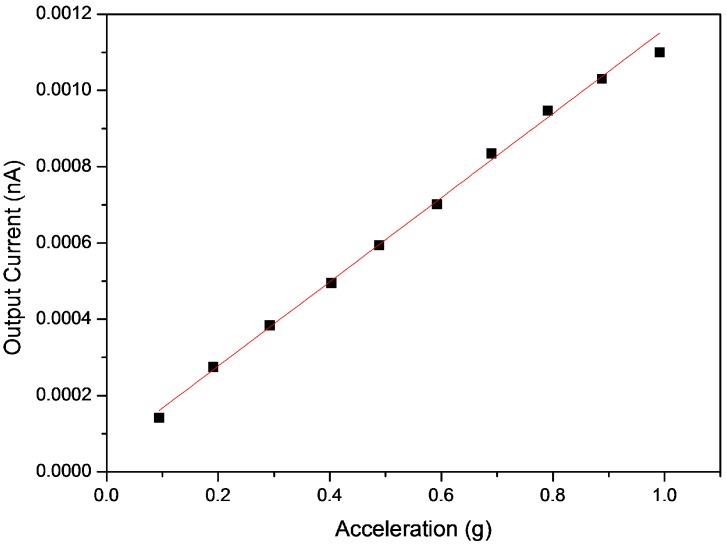
Output current of the fabricated accelerometer and the acceleration of the commercial accelerometer at 100 Hz.

**Figure 10:**
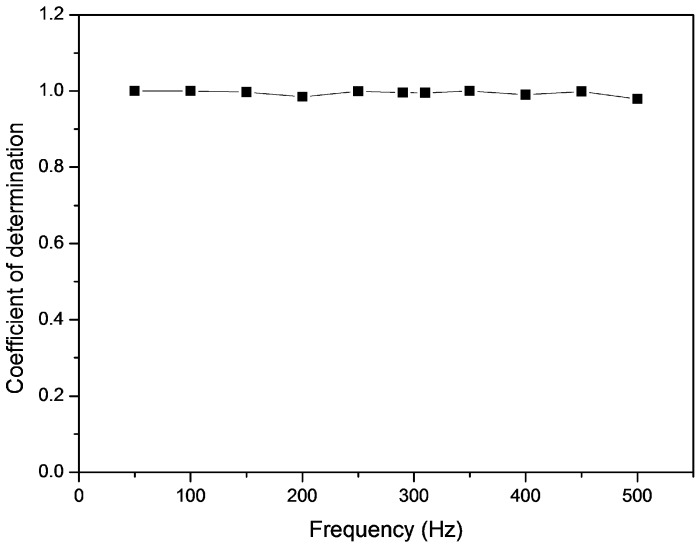
The coefficient of determination (R^2^).

Figure 9 shows the output current of the fabricated accelerometer versus the acceleration of the commercial accelerometer at 100 Hz. A trend line is plotted on the scatter chart which is most likely linear. Moreover, the derivative equation from the trend line is used for calibration. The coefficient of determination (R^2^) was derived from trend analysis which indicates that how well the current data fits the acceleration value ranging from 0 to 1 g. At 100 Hz, the R^2^ between the output current and acceleration was 0.99098 and the analysis was continuously carried out at all operating frequencies. Figure 10 shows R^2^ values between the output current and the acceleration at each frequency in the operating frequency range. The R^2^ ranged from 0.9862 to 0.9998. This indicates that the fabricated accelerometer has consistent sensitivity with frequency change.

## 5. Conclusions

A miniaturized ZnO NW accelerometer was fabricated and its feasibility as an accelerometer was demonstrated. The fabrication process of a polydimethylsiloxane (PDMS) boat was introduced with a Cu substrate. The hydrothermal synthesis method was applied to fabricate ZnO NW arrays on the Cu substrate. To prove the accelerometer performance, comparison with a commercial accelerometer was carried out. The output signal of the fabricated accelerometer increased linearly with the acceleration value of the commercial accelerometer. The fabricated ZnO NW accelerometer has advantages in terms of its simple structure, light weight, low unit cost and possibility of mass production.
